# Promoter hypermethylation as a mechanism for Lamin A/C silencing in a subset of neuroblastoma cells

**DOI:** 10.1371/journal.pone.0175953

**Published:** 2017-04-19

**Authors:** Ines Rauschert, Fabian Aldunate, Jens Preussner, Miguel Arocena-Sutz, Vanina Peraza, Mario Looso, Juan C. Benech, Ruben Agrelo

**Affiliations:** 1 Laboratory of Cellular Signaling and Nanobiology, Instituto de Investigaciones Biológicas Clemente Estable, Montevideo, Uruguay; 2 Epigenetics of Cancer and Aging Laboratory, Institut Pasteur de Montevideo, Montevideo, Uruguay; 3 Bioinformatics Core Unit (BCU), Max Planck Institute for Heart and Lung Research, Bad Nauheim, Germany; Institut d'Investigacio Biomedica de Bellvitge, SPAIN

## Abstract

Nuclear lamins support the nuclear envelope and provide anchorage sites for chromatin. They are involved in DNA synthesis, transcription, and replication. It has previously been reported that the lack of Lamin A/C expression in lymphoma and leukaemia is due to CpG island promoter hypermethylation. Here, we provide evidence that Lamin A/C is silenced via this mechanism in a subset of neuroblastoma cells. Moreover, Lamin A/C expression can be restored with a demethylating agent. Importantly, Lamin A/C reintroduction reduced cell growth kinetics and impaired migration, invasion, and anchorage-independent cell growth. Cytoskeletal restructuring was also induced. In addition, the introduction of lamin Δ50, known as Progerin, caused senescence in these neuroblastoma cells. These cells were stiffer and developed a cytoskeletal structure that differed from that observed upon Lamin A/C introduction. Of relevance, short hairpin RNA Lamin A/C depletion in unmethylated neuroblastoma cells enhanced the aforementioned tumour properties. A cytoskeletal structure similar to that observed in methylated cells was induced. Furthermore, atomic force microscopy revealed that Lamin A/C knockdown decreased cellular stiffness in the lamellar region. Finally, the bioinformatic analysis of a set of methylation arrays of neuroblastoma primary tumours showed that a group of patients (around 3%) gives a methylation signal in some of the CpG sites located within the Lamin A/C promoter region analysed by bisulphite sequencing PCR. These findings highlight the importance of Lamin A/C epigenetic inactivation for a subset of neuroblastomas, leading to enhanced tumour properties and cytoskeletal changes. Additionally, these findings may have treatment implications because tumour cells lacking Lamin A/C exhibit more aggressive behaviour.

## Introduction

Neuroblastoma is an embryonic tumour of the sympathetic nervous system derived from precursor or immature cells, and it accounts for 9%-15% of all deaths in children. Some studies indicate a bimodal age distribution, with one peak at approximately 1 year and the second between 2–4 years [1]. In addition to V-Myc Avian Myelocytomatosis Viral Oncogene Neuroblastoma Derived Homolog gene (MYCN), amplification, chromosome1p deletions, loss of chromosome11q, 17q gains and other imbalances, several gene mutations and epigenetic changes have been reported [2]. It has recently been shown that knockdown of Lamin A/C expression in neuroblastoma cells inhibits cell differentiation and gives rise to a more aggressive and drug-resistant tumour phenotype [3]. Additionally, knockdown of Lamin A/C triggers the development of a human neuroblastoma tumour-initiating cell population with self-renewing features, predisposing this population to a more immature phenotype with enhanced stem cell characteristics [4].

Lamins, which are type V intermediate filaments, are important components of the nuclear lamina. They are divided mainly into A and B(B1 and B2)-type lamins.They provide structural support for the nuclear envelope through a meshwork of filaments that are attached to the inner layer of the nuclear membrane,composing the lamina [5–7].The nuclear lamina contains roles, which confers both nuclear cytoskeletal organization and mechanical stability.It is also important for the non-random positioning of subchromosome domains, the overall organization of chromatin, gene regulation, replication, genome stability, differentiation, and tissue-specific functions [8,9]. Importantly, by interacting with the cytoskeleton, it maintains cellular strength [10, 11]. While B-type lamins are ubiquitously expressed and are essential for cell viability, A-type lamins are mostly found in differentiated somatic cells [12], thus regulating nuclear mechanics [13, 14]. The Lamin A/C gene encodes the A-type lamins A and C, which are isoforms that arise as a result of alternative RNA splicing. Mutations in the Lamin A/C gene have been shown to cause several inherited diseases known as laminopathies [15], ranging from more tissue-specific, such as Emery-Dreifuss muscular dystrophy or cardiomyopathy, to more generalized pathologies, such as atypical Werner Syndrome(WS) and Hutchinson-Gilford Progeria Syndrome (HGPS) [16–21]. HGPS patients express the mutant lamin Progerin generated by a silent point mutation (C1824T) in the Lamin A/C gene. This mutation activates a cryptic splice site and generates a form of lamin A with a deletion of 50 amino acids near the C-terminus. Almost 80% of HGPS patients are heterozygous for this mutation in exon 11 of Lamin A/C [22,23]. HGPS cells exhibit distinct structural and mechanical properties of the nuclear lamina [24,25] and may show disrupted developmental epigenetic programmes [26,27]. Of relevance, HGPS patients do not usually develop neuroblastomas.

The A-type lamin expression has roles in cancer and apoptosis [28]. It is usually reduced or absent in cells with high proliferative potential, e.g., embryonic stem cells (ES cells) or progenitors [29,30], and in a wide range of neoplasias as reviewed in [31]. Considering the different expression levels of Lamin A/C during development, the absence of Lamin A/C could predispose cancer cells towards a more immature phenotype [32].

Importantly, somatic mutations in Lamin A/C are very rare in sporadic neoplasms [32], although a translocation between the tropomyosin–receptor kinase (TRK) and LMNA (LMNA-TRK) in colon cancer and fibrosarcoma has recently been reported [33,34].

Transcriptional inactivation by CpG island promoter hypermethylation is a well-established mechanism for gene silencing in human tumours. During the last decade, using either single gene approach analysis [35–37] or methylome analysis, many genes have been shown to be hypermethylated in neuroblastoma, thus demonstrating prognostic value [38–40].

In addition, we have previously demonstrated that this epigenetic mechanism is responsible for the lack of Lamin A/C in leukaemia and lymphoma [41]. Consequently, we extended our previous studies to neuroblastoma; essentially because of its progenitor nature. Lymphomas and leukaemia cells have a high nuclear-to-cytoplasm ratio, and a scant cytoplasm; two characteristics in common with neuroblastoma cells. These characteristics in cancer progenitors may confer a benefit from silencing Lamin A/C in terms of mechanical properties; as reduced nuclear stiffening may promote neoplastic properties like migration and invasion. In this manuscript, we report for the first time that the Lamin A/C gene undergoes CpG island promoter hypermethylation-associated gene silencing in a subset of neuroblastoma cells. Lamin A/C loss of expression was rescued by DNA-demethylating agents. Furthermore, the reintroduction of Lamin A/C provoked a reduction in cell growth kinetics, migration, invasion, and anchorage-independent growth properties. These observations reinforce the hypothesis that Lamin A/C has a tumour-suppressor role. Of note, the organizational patterns of the cytoskeletal components as restructured: actin type filaments, intermediate filaments and microtubules.

Of importance, the introduction of Progerin induced senescence in an important number of cells, together with cytoskeletal reorganization that differs from that observed following Lamin A/C reintroduction.

Moreover, atomic force microscopy (AFM) [42,43] revealed an increase in Young’s Modulus(E) and cellular stiffening. Of relevance, Lamin A/C silencing mediated by short hairpin RNA (shRNA) in unmethylated cell lines resulted in an increase in cell kinetics, growth, migration, invasion and anchorage-independent growth. In addition, a cytoskeletal organization pattern similar to the one observed in methylated cell lines was induced together with a decrease in Young’s Modulus and decreased cellular stiffening.

Finally, the bioinformatic analysis of a set of methylation arrays for 105 neuroblastoma primary tumuors demonstrated that a group of patients (around 3%) gives a methylation signal in some of the CpG sites located within the the Lamin A/C promoter region analysed by bisulphite sequencing PCR (BSP).

Taken altogether these findings may provide new therapeutic insights into the treatment of neuroblastoma, because tumour cells with silenced Lamin A/C exhibit more aggressive behaviour.

## Results

### Lamin A/C promoter hypermethylation in neuroblastoma cell lines is associated with transcriptional gene silencing

**Fig 1:**
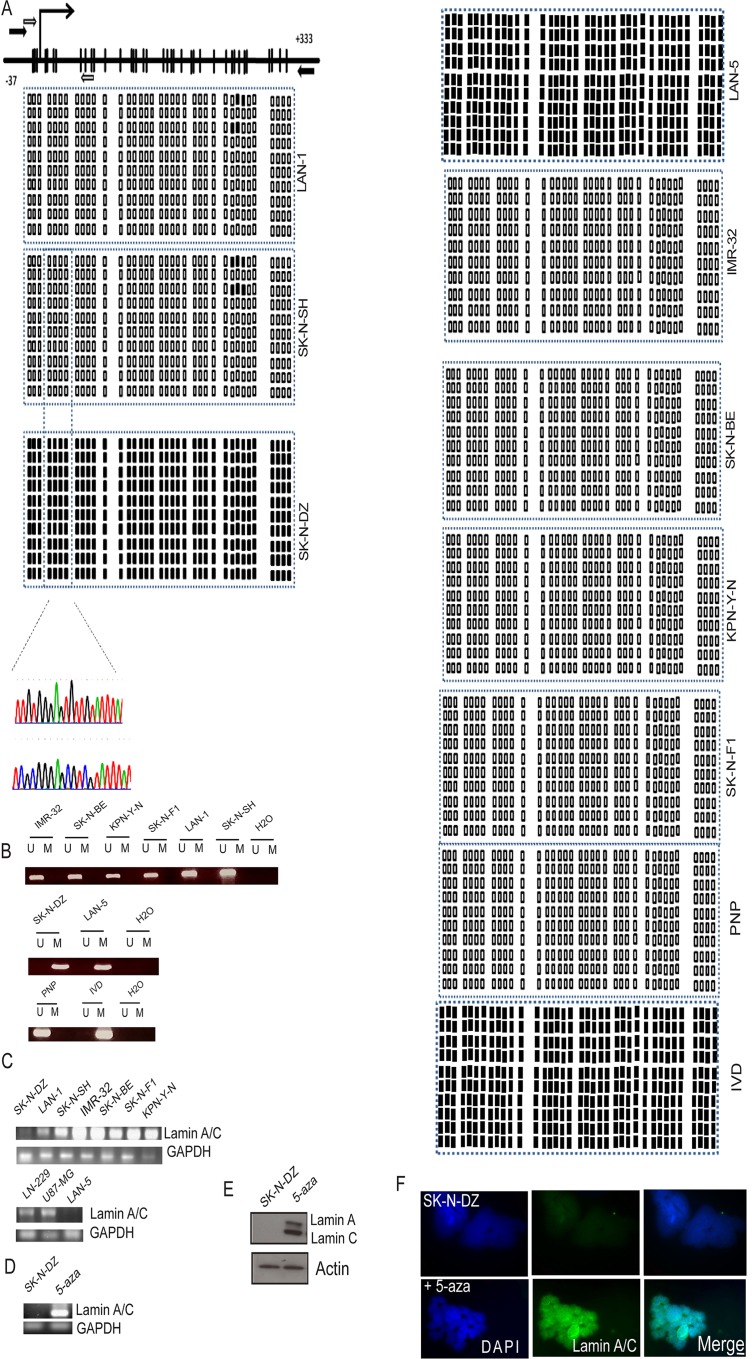
Methylation analysis of the Lamin A/C promoter. **RNA and protein analysis (A)** Schematic of the Lamin A/C CpG Island around the transcription start site (TSS) (long black arrow). CpG dinucleotides are represented as black bars.The locations of bisulphite genomic sequencing PCR primers and methylation-specific PCR primers are indicated as white and black arrows, respectively. The bisulphite sequencing PCR (BSP)results are shown for 10 individual clones for each cancer cell line studied and primary normal human fetal brain neuronal progenitor cells(PNP) cells and IVD αs negative and positive controls, respectively.Two chromatograms are shown to compare one methylated and one unmethylated cell line. **(B)** Methylation-specific PCR (MSP) of the Lamin A/C gene in neuroblastoma cell lines. The presence of a PCR band under lane M indicates methylated genes, while the presence of a PCR band under lane U indicates unmethylated genes. **(C)** RT-PCR analysis of Lamin A/C expression. **(D)** Treatment with the demethylating agent 5-aza reactivated Lamin A/C gene expression in the methylated neuroblastoma SK-N-DZ cell line at the RNA level. **(E-F)** Treatment with 5-aza-2’ deoxycytidine (5-aza) reactivated Lamin A/C gene expression in the methylated neuroblastoma SK-N-DZ cell line at the protein level evaluated by WB and IF, respectively. GAPDH and actin were used as loading controls for RNA and protein expression, respectively. Lamin A/C is observed in green and DNA in blue (DAPI). Scale bar, 10μM.

It has been previously shown that Lamin A/C is silenced by hypermethylation-associated inactivation in human hematologic malignancies [41] (Fig 1A). Because some neuroblastoma cell lines have been shown not to express Lamin A/C, [3] we assessed the methylation status of the promoter-associated CpG island of Lamin A/C in a panel of neuroblastoma cell lines by BSP and methylation-specific PCR (MSP) targeted to the area surrounding its transcription start site (TSS) [41] (Fig 1A, and 1B).

Among the cancer cell lines studied by MSP and BSP, we found that the CpG island was unmethylated in the LAN-1, SK-N-SH, SK-N-BE, KPN-Y-1, SK-N-F1and IMR-32 cell lines (Fig 1A and 1B).Interestingly, we found promoter hypermethylation in the neuroblastoma cell line SK-N-DZ and LAN-5 (Fig 1A and 1B).We used the *in vitro*-methylated DNA (IVD) as a positive control for methylation and primary normal human fetal neuronal progenitor cells (PNP) cells as a negative control.Having noted Lamin A/C promoter hypermethylation in neuroblastoma cell lines, we assessed the association between this epigenetic aberration and putative transcriptional inactivation of Lamin A/C at the RNA and protein levels. First, RT-PCR analysis was performed. The SK-N-DZ and LAN-5 cell lines did not show expression of the Lamin A/C RNA transcripts (Fig 1C and 1D). However, those cell lines with an unmethylated status at the Lamin A/C promoter as well as two glioblastoma cell lines used as positive controls (LN-229 and U87-MG) expressed Lamin A/C transcripts (Fig 1C). Protein expression analysed by Western blotting (WB) and immunofluorescence (IF), for the methylated SK-N-DZ cell line lacked Lamin A/C expression (Fig 1E and 1F). It is well known that demethylating agents can restore gene expression, as observed when we treated the SK-N-DZ cell line with the demethylating agent 5-aza-2’ deoxycytidine(5-aza), which restored the expression of the RNA transcript assessed by RT-PCR (Fig 1D) as well as protein assessed by WB (Fig 1E) and IF (Fig 1F).

### Reintroduction of Lamin A/C in hypermethylated neuroblastoma cancer cell lines induces tumour-suppressor like properties. Introduction of Progerin induces senescence

**Fig 2:**
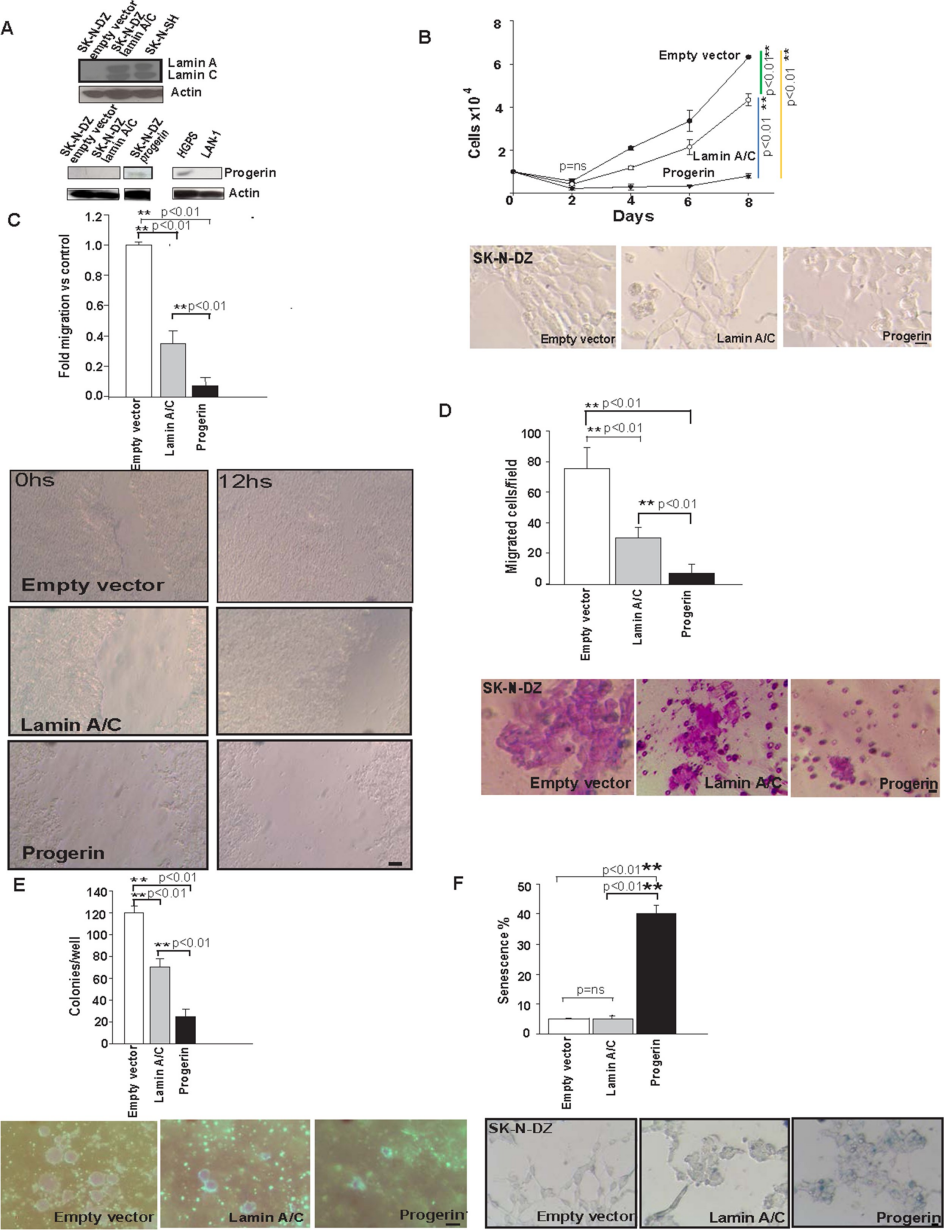
Tumour suppressor like properties after Lamin A/C reintroduction and senescence induction by Progerin introduction in SK-N-DZ cells. **(A)** Lamin A/C expression was monitored by WB for the SK-N-DZ infected cell line either with Lamin A/C (SK-N-DZ-lamin-A/C) or ev (SK-N-DZ-ev). Unmethylated SK-N-SH cells were used as a positive control. Progerin expression was also monitored by WB for SK-N-DZ infected with Progerin (SK-N-DZ-progerin), SK-N-DZ-lamin-A/C and SK-N-DZ-ev The HGPS cell line was used as a positive control, and LAN-1 was used as a negative control. Actin was used as a loading control. **(B)** Cumulative cell numbers of SK-N-DZ-lamin-A/C, SK-N-DZ-progerin, and the SK-N-DZ-ev control. Three independent experiments were performed in triplicate (n = 9) using cells at less than eight passages (n = 9). Statistical significance was assessed by One-way ANOVA test; (**p<0.01). The images above show differential cell growth on day 8 of the experiment and show the different morphologies of these cells. Scale bar, 10μM. **(C)** Wounding assay of confluent cell layers of SK-N-DZ-lamin-A/C, SK-N-DZ-progerin and the SK-N-DZ-ev control. The number of cells that migrated into a delimited wound area after 12h is plotted. Cells in three defined areas per group per experiment were quantified in three independent experiments with three technical replicates. Statistical significance was assessed by One-way ANOVA test; (**p<0.01). Scale bar, 100μM. **(D)** Quantification of the Matrigel chamber migration assay of SK-N-DZ-lamin-A/C, SK-N-DZ-progerin and the S–KN-DZ-ev control. Three independent experiments (n1 = 3) with 3 replicates per experiment (n2 = 9) were performed. Error bars represent the s.d. (n = 9). Statistical significance was assessed by One-way ANOVA test; (**p <0.01). Scale bar, 10μm **(E)** Lamin A/C and Progerin expression have a detrimental effect on anchorage-independent transformation. SK-N-DZ-lamin-A/C formed fewer colonies in soft agar compared to the SK-N-DZ-ev control, whilst SK-N-DZ-progerin formed almost no colonies. The number of colonies per well were counted and plotted. Three independent experiments (n1 = 3) with 3 replicates per experiment (n2 = 9) were performed. Error bars represent the s.d. (n = 9); Statistical significance was assessed by One-way ANOVA test; (**p<0.01). Scale bar, 100μm. **(F)** Quantification of senescence using β-galactosidase staining. Percentage of senescent cells in SK-N-DZ-progerin compared with SK-N-DZ-lamin-A/C and SK-N-DZ-ev. Representative images of β-galactosidase are stained (blue). Three independent experiments with three technical replicates were performed. Error bars represent the s.d. (n = 9) Statistical significance was assessed by One-way ANOVA test; (**p<0.01). Scale bar, 10μM.

It has been proposed that Lamin A/C has tumour-suppressor gene features. Therefore, we assayed the ability of Lamin A/C to function as a suppressor of tumour growth in our model by using the SK-N-DZ cell line. To better understand the effect of Lamin A/C expression in SK-N-DZ cells; they were infected with supernatant containing viral vectors for Lamin A/C expression. First, pBABE-neo-lamin-C or a control empty vector (pBABE-neo-ev) were applied, and stable virus integrations were selected for 15 days with G418 and monitored by WB.Next, Lamin-C-expressing cells were infected with pBABE-GFP-lamin-A or a control empty vector (pBABE-GFP-ev), and stable virus integrations were selected with puromycin.In addition, to explore the possibility that Progerin could rescue the function of the Lamin A/C abrogation, we also infected SK-N-DZ with supernatant containing a viral vector for Progerin expression (pBABE-GFP-progerin). After infection and puromycin selection for 7 days, high-expressing clones were isolated using the green fluorescent protein (GFP) marker.Lamin A/C and Progerin expression were monitored by WB (Fig 2A). Control cells (SK-N-DZ-ev) and Lamin A/C-expressing cells (SK-N-DZ-lamin-A/C) did not express or express Lamin A/C.Unmethylated SK-N-SH was used as a positive control. Regarding Progerin, SK-N-DZ-ev and SK-N-DZ-lamin-A/C did not express Progerin, whereas Progerin-expressing cells (SK-N-DZ-progerin) showed expression. As a negative control, we used the LAN-1 cell line, which displayed no Progerin expression, and as a positive control, a cell line from HGPS showing Progerin expression was utilized (Fig 2A).

Cells stably expressing Lamin A/C SK-N-DZ-lamin-A/C, or Progerin SK-N-DZ-progerin showed markedly slower growth than the control SK-N-DZ-ev cells (Fig 2B). Importantly, the SK-N-DZ-progerin cells showed the slowest growth kinetics (Fig 2B). Moreover, the three different cell lines presented very different morphologies (Fig 2B). To further assess migratory properties, we measured direct migration into an artificial ‘wound’ that was generated in a confluent monolayer culture. Lamin A/C or Progerin expression cell lines (SK-N-DZ-lamin-A/C or SK-N-DZ-progerin) significantly reduced migration and wound closure after 12h compared with control SK-N-DZ–ev cells. Of note, the migration defect was dramatic for cells expressing Progerin (Fig 2C). These results are consistent with those obtained for neuroblastoma cells, in which Lamin A/C was depleted by shRNA [3], as well as the results reported for adult stem cells expressing Progerin [43]. To assess invasiveness, we performed Matrigel invasion chamber assays. Lamin A/C and Progerin expression led to a marked decrease in the number of cells that migrated through this matrix layer, indicating that Lamin A/C decreased cell invasion. Of relevance, Progerin-expressing cells exhibited almost no migration (Fig 2D).

We next investigated the phenotype of Lamin A/C methylated SK-N-DZ cells. To achieve this goal, we performed in vitro assays to examine cellular transformation using either SK-N-DZ-lamin-A/C or control SK-N-DZ-ev cells. SK-N-DZ-lamin-A/C cells formed much smaller numbers of colonies in soft agar than SK-N-DZ-ev controls; whereas almost no colonies were detected for SK-N-DZ-progerin cells (Fig 2E). These observations indicate that the absence of Lamin A/C expression induced anchorage-independent growth of SK-N-DZ cells.

Finally, we performed β-galactosidase staining to evaluate the role of Progerin in the SK-N-DZ cell line. Strikingly, 40% of the SK-N-DZ-progerin cells stained positive for β-galactosidase activity and had a flat morphology, indicating that a large fraction of Progerin-expressing cells displayed signs of senescence, which was not observed for the SK-N-DZ-lamin-A/C or SK-N-DZ-ev cells (Fig 2F).

#### The cytoskeletal pattern is restructured after reintroduction of Lamin A/C in SK-N-DZ cells

**Fig 3:**
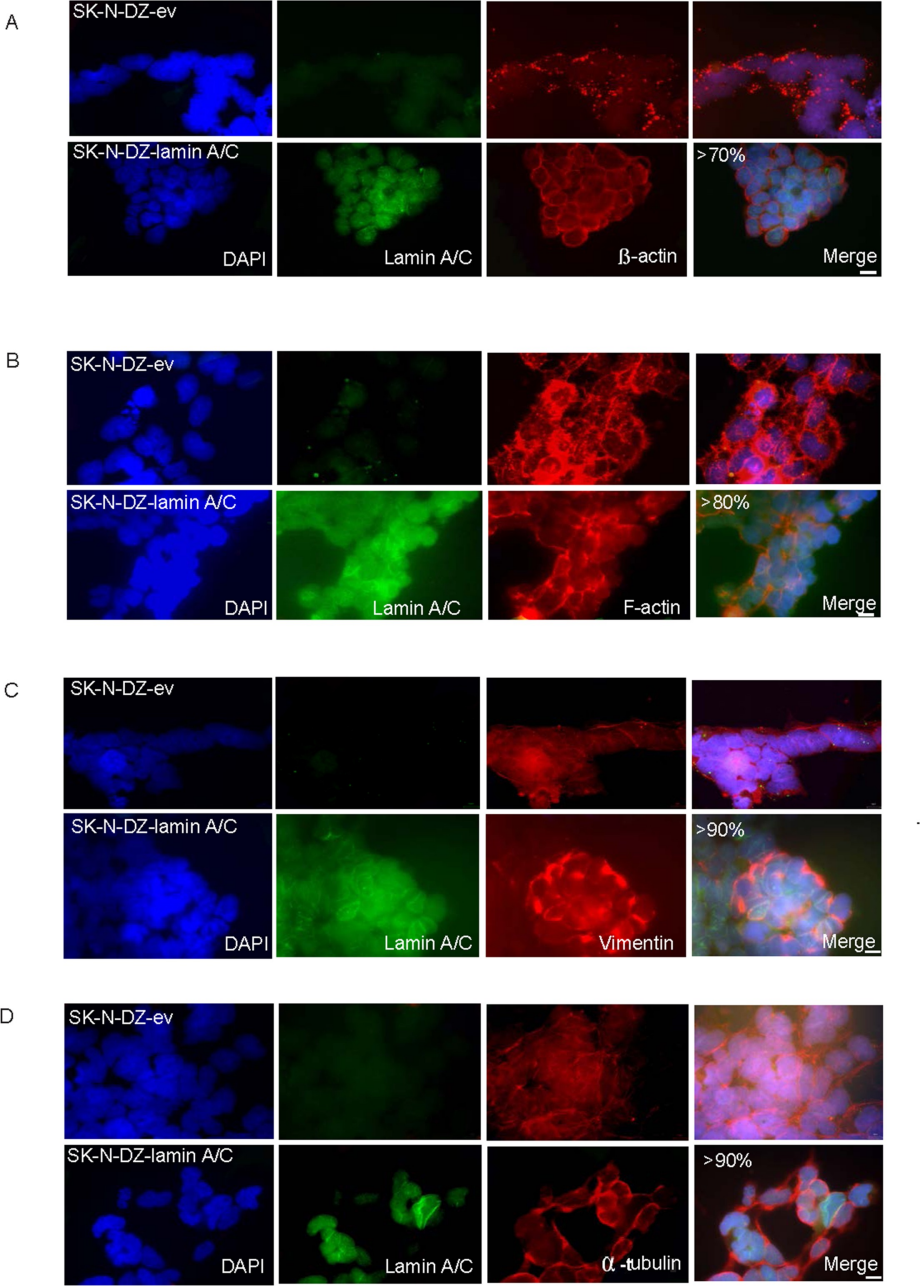
Lamin A/C reintroduction induces reorganizational changes in the different cytoskeletal components. Immunofluorescence staining showing changes in SK-N-DZ-lamin-A/C compared with SK-N-DZ-ev cells in **(A)** β-actin filaments, **(B)** F-actin filaments, **(C)** Vimentin filaments, and **(D)** α**-**tubulin. Lamin A/C is shown in green; β -actin, F-actin, vimentin, and α-tubulin are shown in red.DNA is stained in blue (DAPI). Scale bar, 10μM.

Because of the tight connection between the nuclear lamina and the cytoskeleton, we evaluated whether the lack of Lamin A/C impacts the three main cytoskeletal components. This phenomenon has been previously reported in models such as Lamin A/C -/-mouse embryonic fibroblasts (MEFS) [16]. β-actin is one of six different actin isoforms that have been identified in humans and are involved in cell motility, and one of the two non-muscle cytoskeletal actins. Of relevance, β-actin filaments respond linearly to deformation upon imposing a deforming force [16]. The observed disturbance in structure was restored after Lamin-A/C expression in 70% of the cells (Fig 3A). We also observed a disrupted organization of F-actin filaments, which was rescued by the reintroduction and expression of Lamin A/C in 80% of the cells (Fig 3B). The increased cell motility of SK-N-DZ-ev cells was consistent with the observation of actin organization and increased focal adhesion (Fig 3B). The decreased cell motility was also consistent with the observation of actin reorganization and the decrease in focal adhesions in SK-N-DZ expressing either Lamin A/C or Progerin (Fig 3B).

Importantly, we study the influence of Lamin A/C silencing on intermediate filaments through vimentin. We noted important changes in intermediate filament organization after Lamin A/C reintroduction, presenting a more regular distribution pattern in 90% of the cells. Changes in intermediate networks of filaments have been shown to occur in Lamin A/C -/- MEFS [16] (Fig 3C). Finally, we examined the microtubule organization and, similarly, observed a more disorganized α-tubulin arrangement that changed substantially following Lamin A/C expression. Almost 90% of the cells showed a more regular pattern (Fig 3D). Overall, the cytoskeletal protein patterns were distinct for neuroblastoma cells with methylated Lamin A/C compared with cells in which Lamin A/C was reintroduced.

### Silencing of Lamin A/C in the unmethylated SK-N-SH neuroblastoma cell line leads to a more aggressive phenotype together with changes in cytoskeletal patterns and cellular mechanical properties

**Fig 4:**
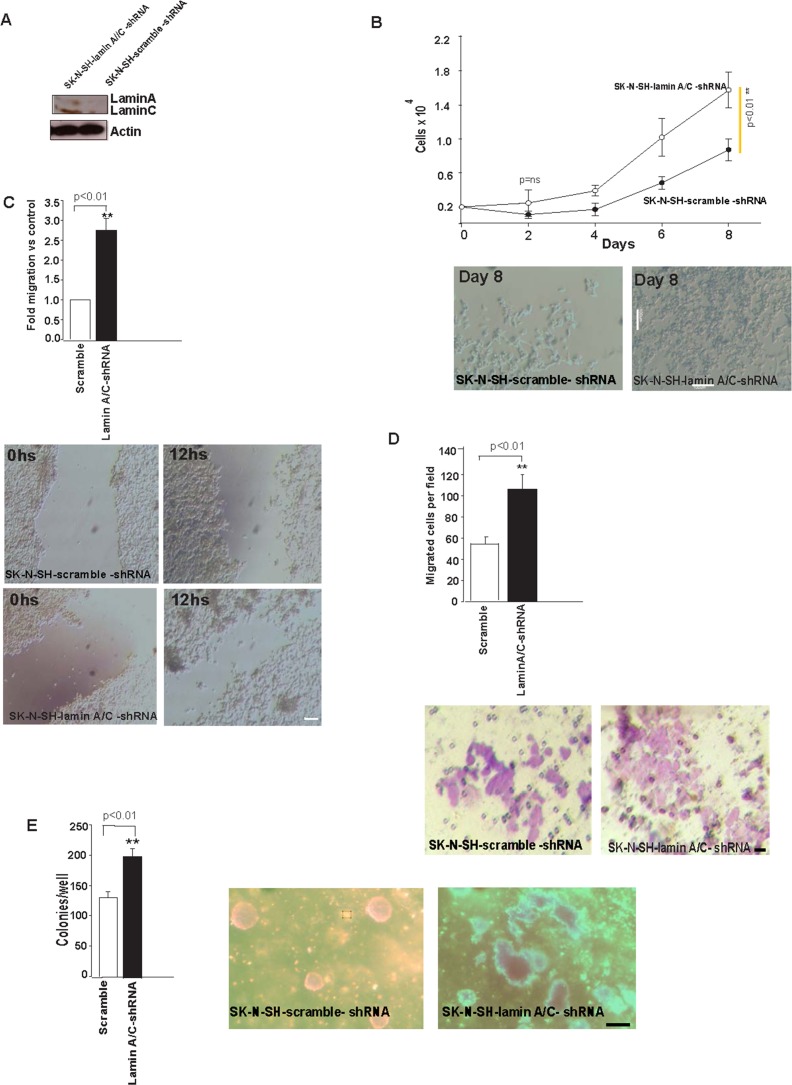
Lamin A/C expression silencing leads to transformation. **(A)** Lamin A/C was monitored by WB after shRNA depletion by SK-N-SH-lamin-A/C-shRNA or SK-N-SH-scramble-shRNA. Actin was used as a loading control. **(B)** Cumulative cell numbers of SK-N-SH-lamin-A/C-shRNA and SK-N-SH-scramble-shRNA, respectively. Three independent experiments were performed in triplicate (n = 9) using cells at less than eight passages, and error bars represent the s.d. Statistical significance was assessed using Student’s t-test;(p<0.01). Representative images showing differential cell growth on day 8 of the experiment. Scale bar, 100μm. **(C)** Wounding assay of confluent cell layers of SK-N-SH-lamin-A/C-shRNA or SK-N-SH-scramble-shRNA.Number of cells that migrated into a delimited wound area after 12h is plotted. Cells in three defined area per group per experiment were quantified in three independent experiments with three technical replicates. Error bars represent the s.d. Statistical significance was assessed using Student’s t-test; (*p<0.01). Representative images; scale bar, 100μM. **(D)** Quantification of the Matrigel chamber migration assay for SK-N-SH-lamin-A/C-shRNA and SK-N-SH-scramble-shRNA. Error bars represent the s.d. (n = 9). Statistical significance was assessed by Student’s t-test; (**p<0.01). Scale bar, 10μM. **(E)** SK-N-SH-lamin-A/C increased the number of colonies in soft agar compared with the SK-N-SH-scramble-shRNA control. The number of colonies per well was counted and plotted. Three independent experiments (n1 = 3) with 3 replicates per experiment (n2 = 9) were performed. Error bars represent the s.d. (n = 9). Statistical significance was assessed using Student’s t-test (**p<0.01). Scale bar, 100μM.

Next, we decided to explore the effect of Lamin A/C silencing in unmethylated SK-N-SH cells in terms of neoplastic properties, cytoskeletal changes and mechanical properties. To pursue this task, we infected the cells with supernatant containing viral vectors (p-GFP-V-RS vectors), (p-GFP-V-RS-lamin-A/C-shRNA) or (p-GFP-V-RS-scramble-shRNA), and selected stable virus integrations with puromycin. After infection and puromycin selection for 7 days, highly expressing clones were isolated using green fluorescent protein (GFP) as a marker. Lamin A/C silencing was evaluated by WB (Fig 4A) which revealed a decrease in Lamin A/C expression in lamin-silenced cells (SK-N-SH-lamin-A/C-shRNA) compared to control cells (SK-N-SH-scramble -shRNA).SK-N-SH-lamin-A/C-shRNA cells had considerably faster growth kinetics than SK-N-SH-scramble-shRNA controls (Fig 4B).

Regarding migration properties, we measured directed migration into an artificial ‘wound’ that was generated in a confluent monolayer culture, as explained above. SK-N-SH-lamin-A/C-shRNA cells showed increased migration and wound closure after 12h, compared with the SK-N-SH-scramble-shRNA control cells. The increased cell motility was also consistent with the observation of actin reorganization and an increase in focal adhesions in F-actin filaments after Lamin A/C silencing (Fig 4C), and with previous results in other neuroblastoma cell lines [3].

We assessed invasiveness by using a Matrigel invasion chamber assay as described in our previous experiments, which revealed a large increase in the number of SK-N-SH-lamin A/C-shRNA cells that migrated through the matrix layer compared with the SK-N-SH-scramble-shRNA cells (Fig 4D), indicating that Lamin A/C silencing increased invasion, which is consistent with previous results obtained in other neuroblastoma cell lines [3].

To investigate the phenotype, we performed in vitro cellular transformation assays using unmethylated lamin SK-N-SH-lamin-A/C-shRNA or SK-N-SH-scramble-shRNA controls. SK-N-SH-lamin-A/C-shRNA formed a greater number of colonies in soft agar than SK-N-SH-scramble-shRNA (Fig 4E); reinforcing our previous data indicating that methylation ofthe Lamin A/C gene promoter increased the capacity for anchorage-independent growth of SK-N-DZ cells.

**Fig 5:**
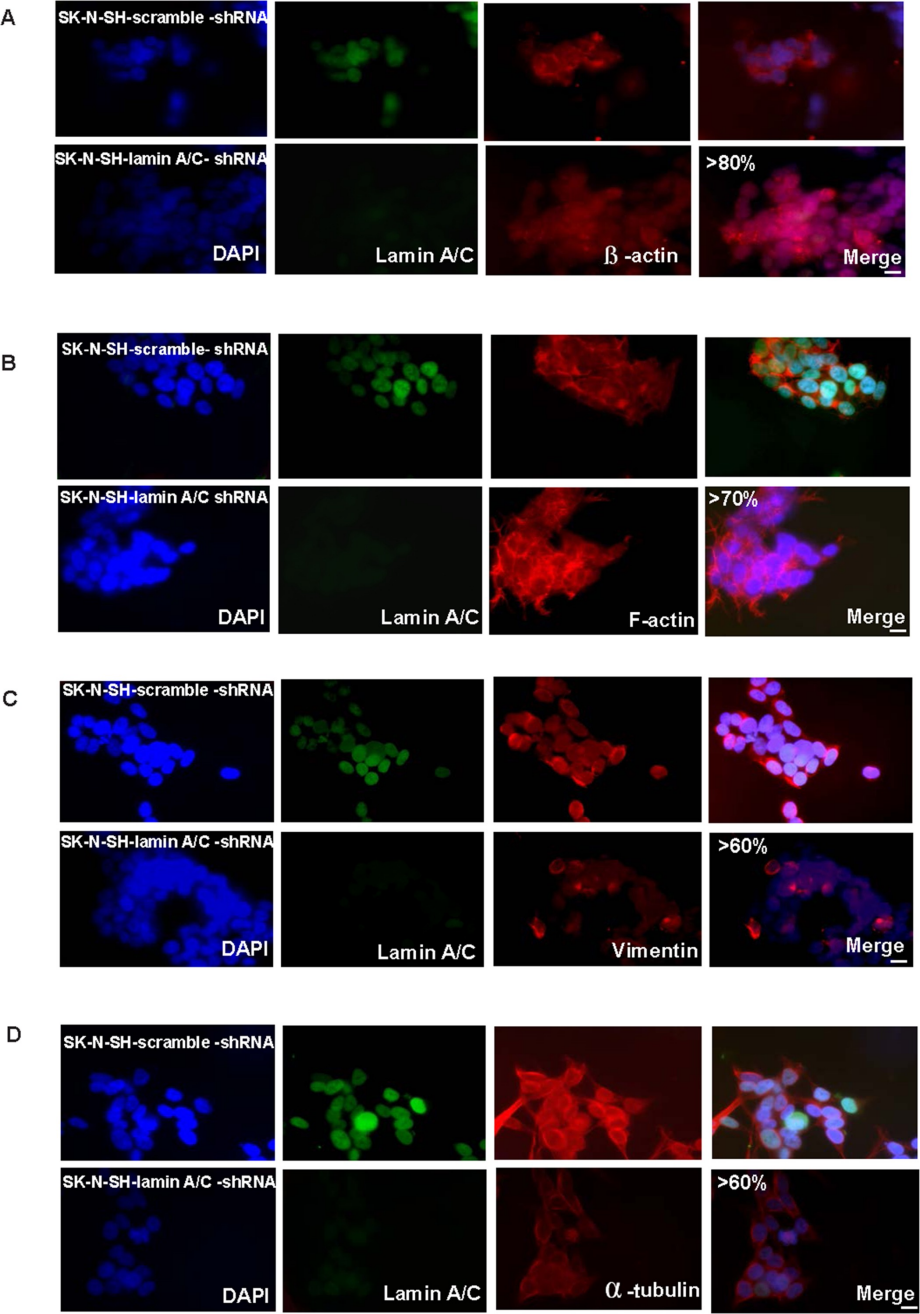
Silencing of Lamin A/C in unmethylated neuroblastoma cells induces changes in different cytoskeletal components. Immunofluorescence staining showing changes in SK-N-SH-lamin-A/C-shRNA compared with SK-N-SH-scramble-shRNA in **(A)** β-actin filaments, **(B)** F-actin filaments. **(C)** Vimentin filaments, and **(D)** α-tubulin. Lamin A/C is shown in green; β-actin, F-actin, vimentin, and α-tubulin are shown in red.DNA is stained in blue (DAPI). Scale bar, 10μM.

Regarding the changes in cytoskeletal components, shRNA-mediated knockdown of Lamin A/C in unmethylated SK-N-SH cells showed reorganization of the three components of the cytoskeleton. They were evaluated through changes in β-actin, F-actin, vimentin and α-tubulin patterns. These changes were observed in 80%, 70%, 60% and 60% of the cells, respectively (Fig 5A–5D).

Microtubules displayed less reorganization, potentially because Lamin A/C silencing did not completely reduce Lamin A/C levels. (Fig 5D). Importantly, the induced cytoskeletal pattern was similar to the one observed in cells with methylated Lamin A/C (Fig 3A–3D and Fig 5A–5D).

**Fig 6:**
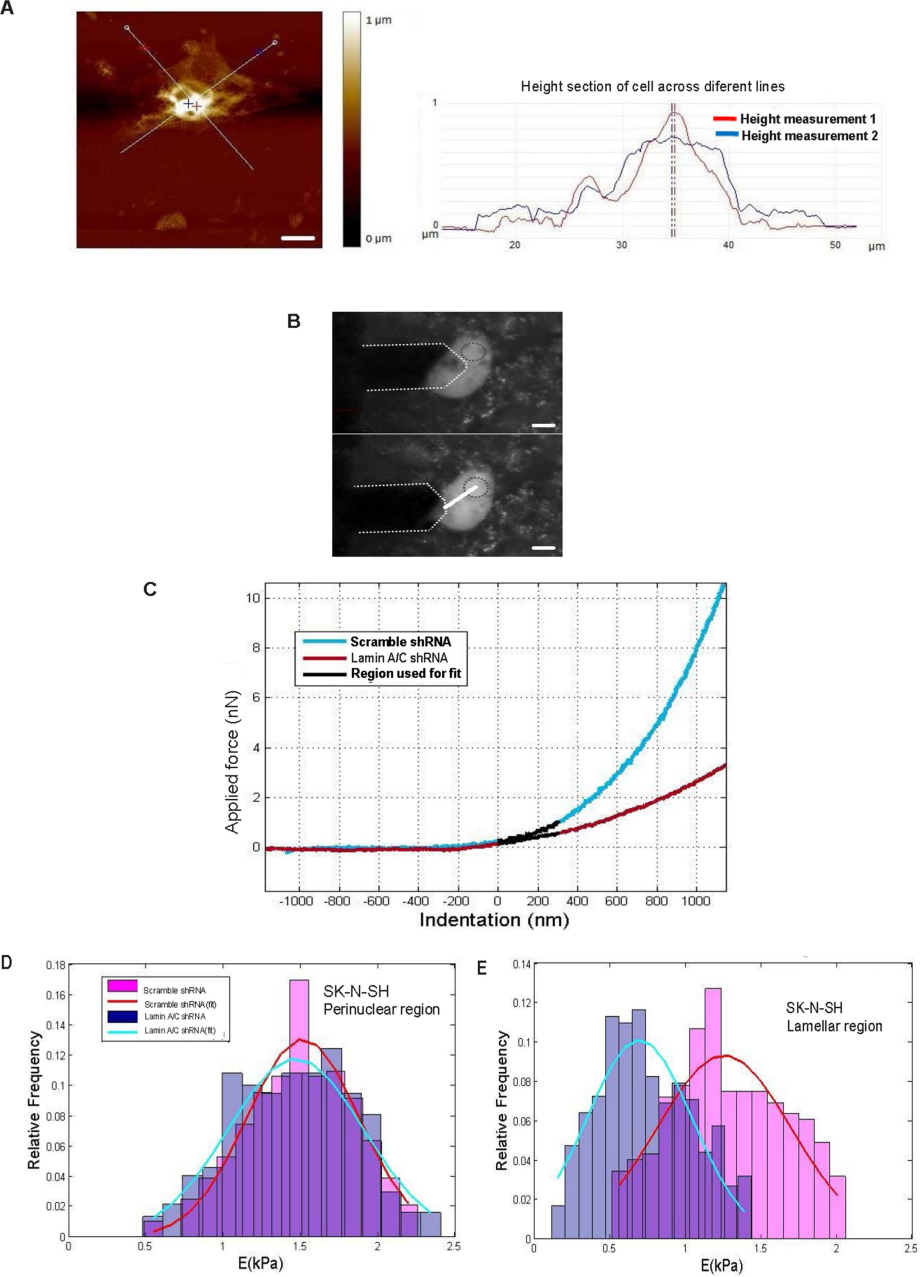
Changes in the mechanical properties of Lamin A/C-silenced unmethylated neuroblastoma cells. **(A)** Representative optical and atomic force microscopy (AFM) images of SK-N-SH-scramble-shRNA for height measurement. The obtained image (150 x 150 μm, 512 x 512 pixel resolution, and 0.2 Hz scan rate) was used to measure the height of the cells. Scale bar, 10μM. In red and blue height profile across different lines from height AFM image are shown. **(B)** AFM tip positioned directly above the lamellar (lower panel) and perinuclear region (upper panel) to show we were capable of carefully selecting the region in which the force curve was obtained. **(C)** Typical force curves from SK-N-SH-lamin-A/C-shRNA or SK-N-SH-scramble-shRNA, and the 300-nm region of the curve used for the polynomial fit. **(D**) Normalized histogram of the data obtained from both groups for the perinuclear region. Each histogram was fitted with a Gaussian curve to obtain the mean value and the standard deviation of Young’s Modulus (E) A Student’s t-test confirmed that there were no significant differences between the two groups. **(E)** Normalized histogram of the data obtained from both groups for the lamellar region. Each histogram was fitted with a Gaussian curve to obtain the mean value and the standard deviation of E. A Student’s t-test confirmed that there were significant differences (**p<0.01).

Finally, we used AFM to evaluate changes in mechanical properties between SK-N-SH-lamin-A/C-shRNA and SK-N-SH-scramble-shRNA control cells. Fig 6A shows a representative image (150 x 150 μm, 512 x 512 pixel resolution, 0.2 Hz scan rate) that we used to measure the height of cells, which was 868+/-242 nm(n = 22). We positioned the AFM tip directly above the lamellar (15μM from the nuclear centre) or perinuclear (9μm from the nuclear centre) regions. We were capable of carefully select the region in which the force curve was obtained (Fig 6B). We defined the lamella as the cytoplasmic region at a distance greater than 10μM from the nuclear centre.

First, the force curves were generated in the perinuclear region of SK-N-SH-lamin-A/C-shRNA and SK-N-SH-scramble-shRNA control cells (Fig 6C). Each curve was fitted to a second-order polynomial equation to obtain a value for Young’s ModulusOnly the first 300 nm of indentation (parabolic region of the curve) were used for the fit to avoid the substrate effect, which is present in the linear region of the curve A normalized histogram of both sets of data and a Gaussian fit for each are shown (Fig 6D).

The values obtained for E in both groups were as follows: E = 1.49± 0.34 kPa for the control group; E = 1.48 ± 0.39 kPa for cells with silenced Lamin A/C. This result is consistent with previous findings in lamin murine knockout MEFS, in which mechanical differences were less significant in this region [44, 45] (Fig 6D).

Second, force curves were generated in the lamellar region because mechanical changes were found in this region in our model. Fig 6C shows typical force curves from cells with and without Lamin A/C expression (SK-N-SH-scramble shRNA and SK-N-SH-lamin-A/C-shRNA) and the 300-nm region of the curve used for the polynomial fit. For the same applied force, the indentation depth was greater for cells with silenced Lamin A/C. This difference indicated that Young’s Modulus would be lower compared with cells expressing Lamin A/C. To confirm this difference, the first 300 nm of indentation from each force curve was fitted to a second-order polynomial equation, and a value for E was obtained. A normalized histogram of both sets of data and a Gaussian fit for each are shown (Fig 6E)The values obtained for E of both groups were as follows: E = 1.28± 0.37 for the control group; E = 0.73 ± 0.30 for cells with silenced Lamin A/C. A Student’s t-test confirmed that the difference was significant (**p<0.01)(Fig 6E).These results clearly showed that silencing of Lamin A/C altered the cellular mechanical properties, reducing the stiffness of the cells.These data are consistent with previous results obtained in Lamin A/C murine knockout MEFS[44,45].

### Cytoskeletal reorganization and mechanical properties of Progerin-expressing SK-N-DZ cells

**Fig 7:**
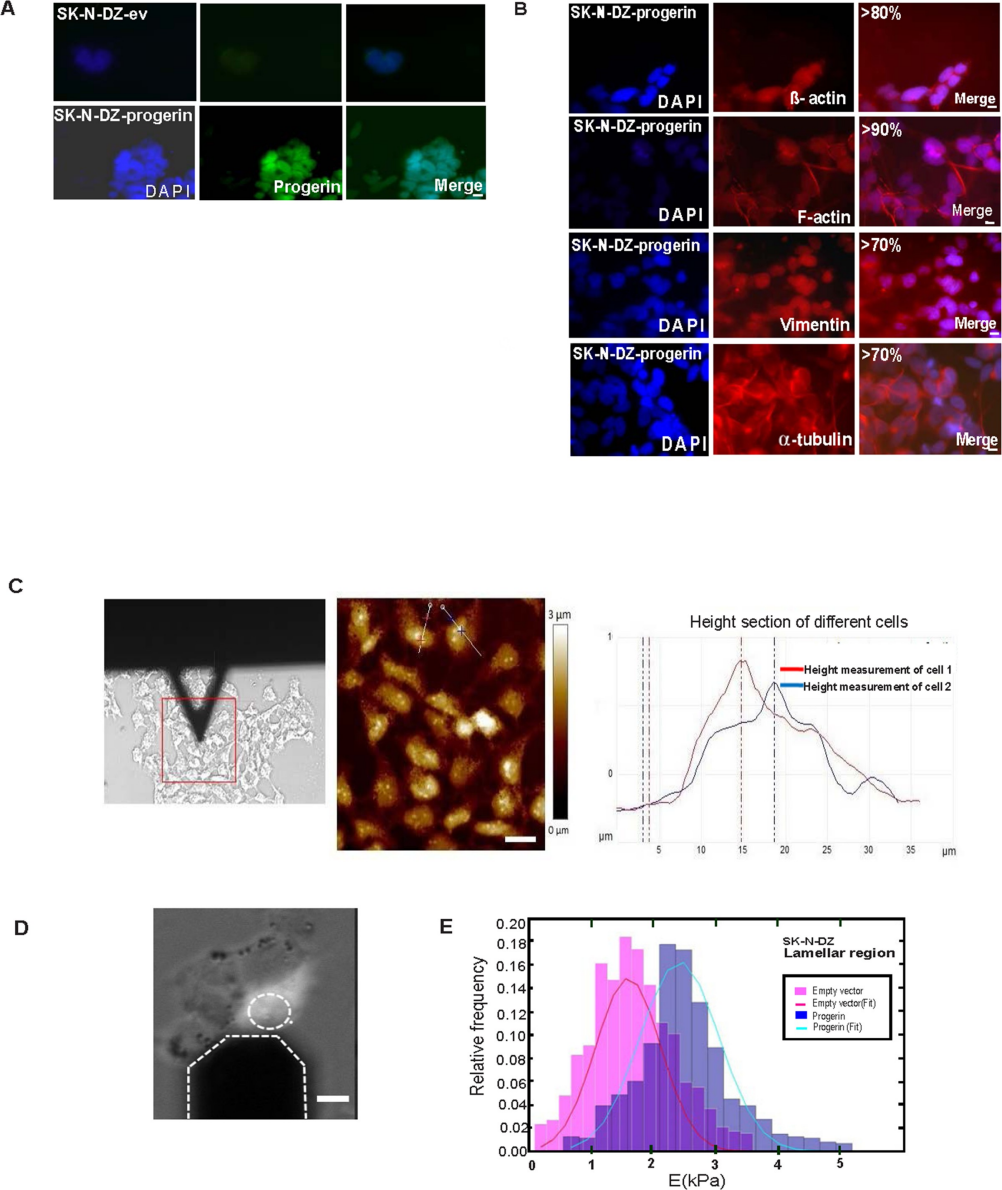
Progerin introduction in SK-N-DZ cells induces changes in cytoskeletal components and mechanical properties. **(A)** Immunofluorescence staining showing Progerin. **(B)** Changes in β-actin, F-actin, vimentin filaments, and α-tubulin after Progerin introduction. Progerin is shown in green; β-actin, F-actin, vimentin, and α-tubulin are shown in red; and DNA is stained in blue (DAPI). Scale bar, 10μm. **(C)** Representative AFM images of height measurement in SK-N-DZ-ev cells. In red and blue height profile across lines in different cells from height AFM image are shown Scale bar, 10μm **(D)**The AFM tip is positioned directly above the lamellar region. Scale bar 10μm **(E)** Normalized histogram of the data obtained from both groups for the lamellar region. Each histogram was fitted to a Gaussian curve to obtain the mean value and the standard deviation of Young’s Modulus. Three independent experiments (n1 = 3) with 3 replicates per experiment (n2 = 9) were performed. A Student’s t-test confirmed that the difference was significant (**p<0.01).

We have observed changes in SK-N-DZ–progerin cells (Fig 7A) compared with the SK-N-DZ–ev control in the three components of the cytoskeleton. However, the cytoskeletal changes observed in SK-N-DZ-progerin cells were different from those observed in SK-N-DZ-lamin-A/C cells. This difference indicates that distinct cytoskeletal changes were produced in cells expressing either Lamin A/C or Progerin. To avoid examining cytoskeletal changes and mechanics due to senescence (which occurs in 40% of these cells), we selected cells that did not present a senescent phenotype. Reorganization was observed in 80%, 90%, 70% and 70% of the cells, respectively (Fig 7B). The decreased cell motility was further consistent with the observed actin reorganization and decrease in focal adhesions in SK-N-DZ expressing Progerin (Fig 7B).

The AFM height of the cells was 1.05 ±0.19 μM (N = 22) (Fig 7C). Force curves were obtained as previously described from the lamellar region for SK-N-DZ-ev and SK-N-DZ-progerin (Fig 7D).

The first 300 nm of each curve was fitted to a second-order polynomial equation, and a value for Young’s Modulus was obtained from each fit. A normalized histogram of both sets of data and a Gaussian fit for each are shown (Fig 7E). The values obtained were as follows: E = 1.67 ± 0.68 kPa for the controls; E = 2.49 ± 0.78 kPa for Progerin. A Student’s t-test confirmed that this difference was significant (**p<0.01). These results clearly show that Progerin significantly increases cellular stiffness and might contribute to the induction of senescence in neuroblastoma cells.

Taken altogether, these results support a contribution of Lamin A/C methylation-mediated silencing in the cytoskeletal and mechanical changes that may contribute to the observed increased tumourigenic properties of cells, inducing increased motility, invasiveness, and transformation properties. Importantly, Progerin induced senescence in an important number of cells, showing a detrimental effect of Progerin on the neoplastic properties of these cells.

### Bioinformatic analysis of Lamin A/C methylation in primary neuroblastoma tumour samples

**Fig 8:**
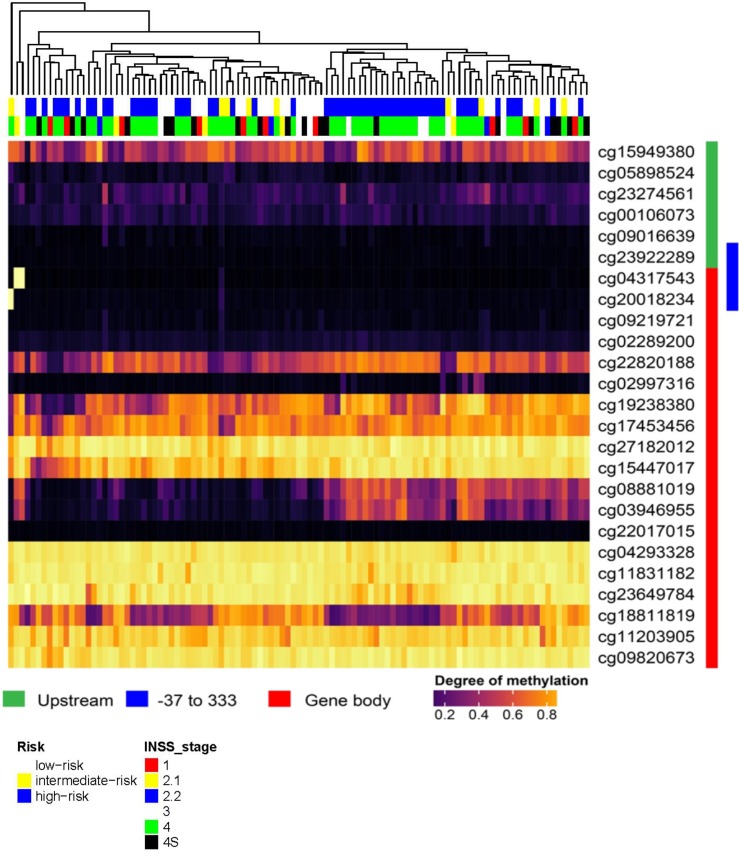
Methylation analysis of the Lamin A/C gene in neuroblastoma patients. Degree of methylation in neuroblastoma tumours at Lamin A/C locus is visualized as colour scale from black(very low methylation) to yellow (fully methylated) in a heatmap, where rows represent CpGs represented on the array (with array probe ID) and columns represent patients. As indicated by coloured bars on the right (green/red), the heatmap illustrates the genomic locations of the Lamin A/C gene from top to bottom, while array locations marked in green represent positions upstream of the TSS, and red marked locations represent the coding sequence In addition, the promoter region -37 to +333 is highlighted in blue. The column tree vizualizes the hierachical clustering on patients. On top of the heatmap clinical parameters: risk (white = low risk, yellow = intermediate risk, blue = high risk) and international neuroblastoma staging system (Inns) stage are given (see colour legend for 6 stages).

In order to investigate whether our previous described findings could be transferred to primary neuroblastoma tumour samples, we analysed a respective set of methylation arrays for 105 patients (GEO dataset GSE 73515). When we accessed the Lamin A/C gene as illustrated in (Fig 8), we found that the majority of patients represented a very low degree of methylation throughout the promoter region of Lamin A/C upstream of the TSS (indicated in green) (Fig 8), while the coding part of the gene was highly methylated in most patients (indicated in red) (Fig 8). In particular, the promoter region of -37 to +333bp described in this manuscript (marked in blue) (Fig 8) is not methylated in the large majority of patients, although three patients give a methylation signal in this region (light yellow patients in the upper left) (Fig 8). Interestingly, all patients cluster into two mayor groups with respect to two CpG sites within the gene body (cg08881019 and cg03946955) that exhibit a dramatic change in the methylation rate (from left to right in the center) (region 2) (Fig 8). Of relevance, this region resides around the TSS of two other Lamin A/C isoforms. As shown in Fig 8, the three patients displaying Lamin A/C promoter methylation in region 1 sites show a low/intermediate risk, while the methylation sites in region 2 show a trend for higher risk in methylated candidates (Fig 8). Moreover, there is an association between the cg08881019 and cg03946955 methylation status and risk (low vs. Intermediate- plus high risk groups); Fisher's exact two-tail test, p = 0.044 (S1 Table).

Patients were sorted according to risk and international neuroblastoma staging system (INSS) stage. DNA methylation was present in fifty one percent of patients. A trend for higher risk in methylated candidates was observed.

Significant association between the methylation status of the two CpGs and risk (low vs. Intermediate- plus high risk patients) was also observed; Fisher's exact two-tail test; p = 0.044.

## Discussion

A-type lamins are essential components of the nuclear lamina and are expressed in most differentiated somatic cells. A much smaller pool of A-type lamins localizes to the nuclear interior [46] to control progenitor cell proliferation [47]. Lamin complexes in the nuclear interior also form part of a diffuse skeleton that is ramified throughout the interior of the nucleus[48].

The connection between the cytoplasm and nucleoplasm might be mediated by the interaction between integral proteins of the INM (the Sun proteins Sun1 and Sun2) and the ONM (the nesprins nesprin-1, nesprin-2, and nesprin-3α) in the luminal space [49, 50]. In the nucleoplasm, Sun proteins interact with Lamin A/C [51], while in the cytoplasm; nesprins may bind to actin [52, 53] and microtubules [54]. This assembly is known as the LINC complex (for LInker of Nucleoskeleton and Cytoskeleton) and establishes a physical connection between the nucleoskeleton and the cytoskeleton [55].

Of fundamental importance, the altered organization of actin-, vimentin- and α-tubulin-based filaments reveals a disturbed interaction of these structures in LMNA -/- MEFS [13, 16]. The physical interaction between nuclear structures (i.e., lamins) and the cytoskeleton causes general cellular weakness and emphasizes a potential key function of lamins in maintaining cellular tensegrity.

Of note, regarding intermediate filaments, a disorganization of the desmin network has been reported in cardiomyocytes lacking Lamin A/C [56] and in cardiac muscle in *Lmna*^N195K/N195K^ animals [57]. These observations further reinforce the presence of additional molecular connections linking the nuclear lamina to the cytoskeleton that remain to be discovered [10, 58].

Mice lacking A type lamins undergo normal embryonic development, while lamin expression increases with differentiation and is only detected in some tissues after birth [59]. In addition, in adult mouse tissues [59], cells with more stem cell properties or highly proliferative potential exhibit lower expression levels of Lamin A/C than somatic cells and tissues [60].

Similarly, ES cells lack Lamin A/C [29, 30] and display upregulated levels during differentiation [61]. Lamin A/C also influences the reprogramming of somatic cells to induce pluripotent stem cells (iPSCs), and artificial silencing of Lamin A/C facilitates iPSCinduction [62]. Importantly, human fibroblasts overexpressing Lamin A/C show a reduced replicative lifespan [63]. Lamin A/C also impairs adipogenic conversion to preadipocytes [64]. These observations could be partly explained by changes in Lamin A/C interactions with chromatin during differentiation, which play a role in lineage determination and the loss of phenotypic plasticity.

Regarding cancer, a large number of studies have shown that Lamin A/C expression is reduced in transformed cells. For example, a low level of lamin expression has been detected in many prostate, breast, colon, and ovarian cancers, and it is usually associated with a worse prognosis, as reviewed in [65]. However, in some colon cancer and prostate cell lines, Lamin A/C expression seems to confer a more aggressive phenotype [66, 67]. One possibility is that tumours with high expression levels of Lamin A/C may still contain a subpopulation of cancer stem cells (CSCs) with low Lamin A/C expression. It is noteworthy that Lamin A/C expression is absent in stem cells from the gastrointestinal tract (GI) in a mouse model with a specific lamin deletion that exhibits enhanced polyp growth in response to an oncogenic stimulus [68]. Finally, downregulation of A-type lamins in cancer cells results in increased nuclear deformability. This property can facilitate the transit of cells through narrow constrictions such as the interstitial space or during intra- and extravasation and passage through narrow capillaries [65]. Migration-induced damage in these cells associated with mechanical stress may increase nuclear fragility and rupture [69]. These phenomena could influence chromatin organization, increasing DNA translocations, genomic instability, and finally promoting cancer progression [70].

In agreement with these studies, in leukaemia and lymphoma, aberrant transcriptional silencing by CpG island promoter hypermethylation is an important mechanism underlying the loss of Lamin A/C expression and is associated with a poor outcome in diffuse large B-cell lymphomas (DLBCLs) [41]. The lack of Lamin A/C expression in malignant B- and T-lymphocytes may reflect blockade of the differentiation pathway in these cells [32].

In this work, we extend our previous studies to neuroblastoma. First, because of its progenitor nature, recent studies suggest that these cells are derived from neuroblasts [71]. Second, primary neuronal progenitors are derived from the subgranular zone of the dentate gyrus, and the subventricular zone of the lateral ventricle in rats lack Lamin A/C [72]. Third, it has recently been demonstrated that neuroblastoma cells in which Lamin A/C was artificially silenced showed increased tumourigenic properties[3], with a subpopulation exhibiting stem cell characteristics[4]. Finally, cells in which the Lamin A/C gene was found to be methylated (lymphomas and leukaemia) have a high nuclear-to-cytoplasm ratio and very little cytoplasm like neuroblastoma cells. Such a high nuclear-to-cytoplasm ratio in cancer progenitors may confer a benefit from silencing Lamin A/C in terms of mechanical properties. Reduced nuclear stiffening may promote neoplastic properties such as migration and invasion.

Here, we report for the first time that CpG promoter hypermethylation is a mechanism underlying Lamin A/C silencing in a subset of neuroblastoma cells. Some cell lines are methylated and others are not might reflect a less differentiated phenotype in the former populations. SK-N-DZ is derived from a bone marrow metastasis from a child with poorly differentiated embryonal neuroblastoma with a high level of methylation.

Moreover, the reintroduction of Lamin A/C into transformed cell lines with Lamin A/C deficiency due to hypermethylation induced a decrease in cell growth kinetics, migration invasion, and production in colony formation together with cytoskeletal reorganization (Fig 2 and Fig 3). It has been suggested that the increased rate of cell proliferation and the decreased ability to undergo cell cycle arrest observed in LMNA -/- MEFS may be due to the destabilization of phosphorylated retinoblastoma (pRb) and its proteasomal degradation [73, 74], which suggests that depletion of Lamin A/C could promote proliferation [75–77]. Lamin A/C can tether pRb through LAP2alpha protein, promoting hypophosphorylation of Rb and a delay in cell cycle entry [78]. Moreover, pRb is a key factor in HGPS pathogenesis, and its modulation can ameliorate premature ageing [79]. Furthermore, Lamin A/C influences pathways that are known to participate in both tumour suppression and tumour progression, such as TGF-β, and Notch.

In addition to reprogramming, these signalling pathways are important for stemness and have been previously implicated in stem cell phenotypes in cancer, as reviewed in [80]. Future work should explore the roles of these pathways in neuroblastoma cell lines with methylated Lamin A/C. For example, it will be important to address the potential contribution to pRb destabilization in these cells [76].

However, in the present study, we focused on the contribution of the observed cytoskeletal organization and cellular mechanical changes as downstream mechanisms, which may contribute to a more aggressive phenotype in neuroblastoma cells with a hypermethylated Lamin A/C gene.

Similarly, pluripotent stem cells lacking Lamin A/C have a less developed cytoskeleton compared with fibroblasts expressing the protein. Moreover, during the reprogramming of MEFs to iPSCs, the cytoskeleton is remodelled to a less developed state in the resulting iPSCs that lack Lamin A/C [81]. Importantly, the shape of the nucleus is a quantifiable discriminant of the mechanical properties of the cytoskeleton of various stem cell types [82]. Finally, the cytoskeleton suffers changes following the differentiation of certain neuroblastoma cell lines [83].

Lamin A/C deficiency causes impaired mechanotransduction and decreased mechanical stiffness [13,84]. These alterations may be explained, in part, by modifications of the connections between the nucleoskeleton and the cytoskeleton, but not as a consequence of changes in the expression of cytoskeletal proteins.An array analysis comparing the gene expression of LMNA -/- and LMNA +/+ MEFs did not show any changes in the expression of genes encoding cytoskeletal proteins or have any direct effect on the cytoskeleton or cytoskeletal structures [44].

The observed patterns of actin, intermediate filaments and microtubule networks were restructured following the reintroduction of Lamin A/C in neuroblastoma cells with a hypermethylated Lamin A/C gene promoter.

The observation that methylated cells usually express a minimum amount of protein [85] may explain why the connections between the cytoskeleton and nuclear lamin were not completely disrupted.

Our results suggest that in addition to the reported ultrastructural anomalies in the nuclear envelope in many laminopathic tissues, disease model cell cultures, and LMNA -/- MEFs, abrogation of Lamin A/C expression by promoter hypermethylation affects the cytoskeletal organization. It may also contribute to the observed increase in neoplastic properties.

When Lamin A/C was knocked down by shRNA in unmethylated cells, the downregulation of Lamin A/C induced an increase in cell growth kinetics, migration and invasion, and colony formation. Moreover, the observed cytoskeletal pattern was similar to that in the methylated SK-N-DZ cell line.

In addition, the decreased stiffening and Young’s Modulus observed in silenced cells compared with the control was found in the lamella (Fig 6E) but not in the perinuclear region, which showed no significant differences (Fig 6D). This result is consistent with previous findings obtained in LMNA -/- MEFs, in which minimal differences were found in the perinuclear region [44]. These results may suggest that reduced stiffness in Lamin A/C-depleted cells may be responsible, in part, for the observed increase in neoplastic properties such as migration and invasion potential [86].

Furthermore, when Progerin was introduced into methylated Lamin A/C cells, it induced tumour suppressor properties, cytoskeletal reorganization, and, remarkably, senescence in a large number of cells. In addition, as measured by AFM, these cells showed an increase in stiffening and Young’s Modulus compared with the ev-control cells.

Consistent with our results, the nuclear properties of Progerin-transfected Xenopus nuclei showed an increased Young’s modulus that rendered the cells less elastic [87]. Furthermore, melanoma cells transfected with Progerin had a stiffened nucleoskeleton and impaired invasion as compared with the mock control cells [88].

Thus, our results could have clinical implications for HGPS patients who rarely develop neuroblastomas. In addition, naive stem cells from HGPS patients express low levels of Progerin *in vivo* [89], which may lead to stem cell exhaustion [90]. The expression of Progerin in iPSC-derived neurons induces multiple ageing-related markers and characteristics[91].

Neuroblastoma development in adults is very rare but may occur [92]. Because cells accumulate small amounts of Progerin in samples of skin in elderly patients [93], it is tempting to speculate that the same occur sin neuroblasts and that Progerin might protect the older population from this type of tumour, in particular because the subventricular zone (SVZ) maintains the ability to produce neuroblasts in the adult human brain [94–96].

Finally, we extended our previous described findings to primary neuroblastoma tumour samples, and analysed a set of methylation arrays for 105 patients.Interestingly some patients(around 3%) displayed a methylation signal in some of the CpG sites located within the Lamin A/C promoter region analysed by BSP (Fig 1A and Fig 8). Of interst all patients cluster into two mayor groups with respect to two CpG sites within the gene body (cg08881019 and cg03946955) that exhibit an impressive change in the methylation rate (Fig 8). This finding warrants further investigation studies, as this region (region 2) resides around the TSS of two other Lamin A/C isoforms. In addition the three patients displaying Lamin A/C promoter methylation in region 1 sites show a low/intermediate risk, while patients with methylated sites in region 2 show a trend for higher risk (Fig 8 and S1 Table).

In accordance with the international neuroblastoma staging system (INSS)(Fig 8), the three patients with Lamin A/C methylation in some of the CpG sites located within the Lamin A/C promoter region analysed by BSP (Fig 1B and Fig 8); were diagnosed with stages 2.1/2A, 3 and 4 (Fig 8). For patients diagnosed with these stages, lymph nodes enclosed within the tumor (Stage 2.1/2A), nearby lymph nodes (Stage 3), or distant lymph nodes (Stage 4); may contain neuroblastoma cells. This observation may be consistent with our previous results showing that of lack of Lamin A/C favours migration and invasion.

Because silencing of Lamin A/C in neuroblastomas has important mechanical consequences for the cell, low intensity therapeutic ultrasound emerges as a promising therapeutic option for these tumours [97–100]. By studying the overall frequency response of single cell systems to these mechanical stimuli in breast cancer cell lines versus control breast cells(bolstered by finite element method analysis),it has been shown that lower frequencies are needed to ablate cancer cells compared with controls[101]. In this context, it will be interesting to examine whether lower ultrasonic frequencies could have a major impact on neuroblastoma cell destruction in cells with hypermethylated Lamin A/C promoter, which displays more aggressive behaviour[102–104].

Aditionally, the US Food and Drug Administration has approved the use of the demethylating agent 5-azacytidine at low doses for the treatment of myelodysplastic syndrome, based on the capacity of the drug to reactivate CpG island hypermethylated genes [105–107]. Thus, Lamin A/C may represent an effective target gene for such a drug to achieve its demethylation and reactivation. Future studies are warranted from the translational perspective to further evaluate the potential of Lamin A/C methylation status in primary neuroblastomas as a tumour marker.

## Conclusions

Neuroblastoma cells with silenced Lamin A/C display more aggressive behaviour.

## Materials and methods

### Human cell lines

The human neuroblastoma cells LAN-1, SK-N-SH, SK-N-DZ, SK-N-BE, KPN-Y-1, SK-N-F1, LAN-5 and IMR-32 and the glioblastoma cells LN-229 and U87-MG examined inthis study were obtained from the American Type Culture Collection (Rockland, MD, USA) or the German Collection of Microorganisms and Cell Cultures, Braunschweig, Germany) and have been described elsewhere. The HGPS AG06917 cell line was obtained from Coriell Repositories (NJ,USA). Human Fetal Brain Primary Dopaminergic Neuronal Precursor Cells were obtained from Applied Biological Materials (ABM) Inc (Richmond, BC, Canada). The cell lines were maintained in appropriate media and treated with 1 μM 5 aza-2’deoxycytidine (5-aza) (Sigma) for 48h to achieve demethylation, as previously described [41].

### DNA methylation analysis of the Lamin A/C gene

We established the Lamin A/C CpG island methylation status by PCR analysis of bisulphite-modified genomic DNA. First, the methylation status was analysed by bisulphite genomic sequencing of both strands of the CpG Island. The primers used have been described elsewhere [41]. The second analysis utilized methylation-specific PCR (MSP) with primers specific for either the methylated or modified unmethylated DNA. The primer sequences for the unmethylated reaction and the methylated reaction have been previously described [41].

### Lamin A/C RNA and protein analysis

RNA was isolated using TRIzol (Life Technologies, Gaithersburg, MD). RNA 2 *μ*g was reverse-transcribed using SuperScript II reverse transcriptase (Gibco/BRL, Barcelona, Spain) and amplified with specific primers for Lamin A/C as described previously [41]. RT-PCR primers have been described [41]. GAPDH was used as an internal control. Total cell extracts were prepared with radioimmunoprecipitation assay buffer [108]. Nuclear cell extracts and western blotting were performed as previously described [106]. Antibodies were as follows: Lamin A/C diluted 1:1000 (rabbit anti-Lamin A/C (H-110), SantaCruz). Progerin diluted 1:1000(mouse anti-Progerin (Mab13A4), AlexisBiochemicals).

### Immunofluorescence analysis

SK-N-DZ and SK-N-SH cell lines were grown on Roboz slides (Cell Point Scientific, Gaithersburg, MD, USA). Immunostaining was performed as previously described [108]. Briefly, the cells were fixed for 10min in 4% paraformaldehyde in phosphate-buffered saline (PBS), permeabilized for 5min in 0.1% Na-citrate/0.5% Triton X-100 and blocked for 30min in PBS containing 5% bovine serum albumin and 0.1% Tween-20. The antibodies and dilutions used for the analysis were as follows: vimentin diluted 1:200 (mouse anti-vimentin (Ab8978, Abcam), β-actin at 1μg/ml (rabbit anti-β-actin (Ab8227), Abcam), α-tubulin diluted 1:500 (mouse anti-alpha Tubulin (T 9026), Sigma-Aldrich), Lamin A/C diluted 1:250(mouse anti-LaminA/C (Ab4789), Abcam), Progerin diluted1:10(mouse anti-Progerin(Mab13A4), AlexisBiochemicals). Secondary antibodies have been previously described [108]. Vectashield (Vector Laboratories, Burlingame, CA, USA) was used as the imaging medium. DNA was stained with 4’, 6’-diamidino-2-phenylindole (DAPI). Images were captured either with an OLYMPUS IX81 fluorescence microscope (Olympus Center Valley, PA, USA) or a LEICA TCS-SP5-DMI6000 confocal microscope (Leica Microsystems, Buffalo Grove, IL, USA) and then analysed using Image-Pro Plus software (Media Cybernetics, Inc.) or LAS AF-Lite (Leica Microsystems, Buffalo Grove, IL, USA). Colour levels were adjusted in Photoshop CS6 (Adobe). (Adobe Systems Incorporated CA, USA). Three independent experiments (*n*_1_ = 3) with 3 replicates per experiment (*n*_2_ = 9) were performed. Three hundred cells were counted per condition in each experiment.

### Phalloidin staining

To analyse F-actin filaments, cells were incubated with Texas Red-X phalloidin (Molecular Probes, Eugene, OR, USA) according to the manufacturer’s protocol. Briefly, the cells were fixed for 10min in 4% paraformaldehyde in PBS, permeabilized in Na-citrate/0.5% Triton X-100, blocked for 30min with 1% bovine serum albumin in PBS and incubated with Texas Red^-^X phalloidin (1:100 dilution) for 30min. DNA was stained with 4′,6-diamidino-2-phenylindole-dihydrochloride(DAPI).

### Colony-forming assay in soft agar

Cells (2.0×10^4^) were plated in 0.36% agar in Dulbecco's Modified Eagle’s Medium (soft agar medium) per 60-mm dish on top of a 0.72% hard agar layer. Cultures were reefed by addition of 3ml soft agar medium after 1–2 weeks. After 21 days, the colonies were counted using an inverted microscope. The results represent three independent experiments that were performed in triplicate (*n* = 9) and error bars represent the s.d.

### Wound healing assays

An artificial wound was generated with a 10-μl pipette tip on confluent monolayers of the neuroblastoma cell lines SK-N-SH-lamin-A/C-shRNA and SK-N-SH-scramble-shRNA control cells or SK-N-DZ-lamin. SK-N-DZ-progerin and SK-N-DZ-ev, respectively, were grown in six-well culture plates in serum-containing medium. Photographs were obtained at 0h and 12h, respectively. Analysis of wound closure was calculated by counting the cells per μm^2^ of wound area at 12h. The results represent three independent experiments that were performed in triplicate (*n* = 9), and error bars represent the s.d.

### Two-chamber migration and invasion assays

Cell invasion was determined using the BD Biocoat Matrigel Invasion Chamber (8μm pore size, BD Biosciences, San Jose, CA, USA) invasion assay (membrane coated with a layer of Matrigel extracellular matrix proteins), according to the manufacturer's instructions. Cells (5.0 × 10^4^) were seeded in serum-free medium in the upper chamber, and 10% foetal calf serum was used as a chemoattractant in the lower chamber. After 18h, the cells in the upper chamber were carefully removed using cotton buds, and the cells at the bottom of the membrane were fixed in 4% paraformaldehyde in PBS and stained with crystal violet (0.2%). Quantification was performed by counting the stained cells. Three independent experiments were performed in triplicate (*n* = 9), and error bars represent the s.d.

### Virus production and infection of neuroblastoma cells

The human pBabe-puro-GFP-wt-lamin-A and pBabe-puro-GFP-Progerin vectors have been described [109], and the control was pBabe-puro-GFP. The human pBabe-neo-lamin-C was used, and pBABE-neo control has been described [110].

LMNA 29-mer shRNA constructs in retroviral p-GFP-V-RS vectors and a scramble control (Origene, Rockville, MD, USA) were used for transfection. The construct used targets a common Lamin A/C gene sequence.

Retrovirus production was performed as previously described [111,112]. Briefly, producer Plat-A cells were transfected using Effectene (Qiagen). Viral supernatants were collected after 48h and used for infection. Cells were infected for 48, and after 24h selected with G418 and/or puromycin until colonies appeared. Colonies were picked using cloning cylinders (Millipore, Billerica, MA, USA) and expanded. Cells were assessed for protein expression by WB. For kinetic studies, 0.2x10^4^or 10^4^ cells, were plated in twelve-well plates in triplicate, and the cell numbers were determined every 2 days using a Neubauer chamber cell counter (Roche Diagnostics, Mannheim, Germany). Three independent experiments were performed in triplicate (*n* = 9) using cells at less than eight passages, and error bars represent the s.d.

### Senescence analysis

β-gal staining was performed using the Senescence β-Galactosidase Staining Kit (Number 9860, Cell Signaling Technology, Danvers, MA, USA) according to the manufacturer’s instructions. Three independent experiments were performed in triplicate (*n* = 9) using cells at less than eight passages, and error bars represent the s.d.

### AFM images

AFM images from fixed SK-N SH and SK-N-DZ cells were obtained with an atomic force microscope (BioScope Catalyst, Bruker). Cells were grown on Roboz slides (Cell Point Scientific, Gaithersburg, MD, USA), fixed for 10 min at 4°C in 1% paraformaldehyde, washed three times with MilliQ ultrapure water, and imaged with a silicon nitride probe (SNL-10, Bruker, Cantilever A) (Bruker Camarillo, CA, USA). The pyramidal probe had a radius of 2 nm and was attached to a 120-μm-long triangular cantilever with a spring constant of 0.35 N/m, according to the manufacturer’s instructions (Fig 7C).

### Nanomechanical properties and statistical methods for data analysis

Elasticity measurements were obtained using the nano-indentation method with an atomic force microscope (BioScope Catalyst)(Bruker, Camarillo, CA USA), as described in [113]. The cells were cultured on Roboz slides (Cell Point Scientific, Gaithersburg, MD, USA) placed into an inverted microscope (Olympus IX81)(Olympus,Center Valley, PA, USA) coupled to the AFM.Before conducting the AFM experiments, the cell medium was changed from DMEM (1X) + GlutaMax with 10% serum and antibiotics to DMEM (1X) + GlutaMax. All AFM measurements were conducted within 1 h after insertion of the AFM head.

The probe used (OBL-10, Bruker, CantileverA) (Bruker, Camarillo, CA, USA) consists of a pyramidal Silicon Nitride tip with a radius of 30 nm attached to a 70-μm-long rectangular cantilever with a spring constant of 0.03 N/m, according to the manufacturer’s instructions.

We used 20X and 40X magnification eye pieces to position the cantilever tip directly above the region of interest (perinuclear or lamellar depending on the case) of an immobilized cell. Once the tip was in contact with the sample, a low resolution 500 nm x 500 nm image was obtained in contact mode. The “point-and-shoot” application of the AFM software (Nanoscope software) (Bruker, Camarillo, CA USA) was used to select a series of points over the obtained image. Force curves with an indentation-and-retraction rate of 6μm/s were obtained in force calibration mode at the selected points. The force-curve frequency was set to 1 Hz to minimize hysteresis and drag force [114]. At least 10 force curves were captured for each point, and between 500 and 1000 force curves were captured from each cell.

The applied force was calculated using Hooke’s law, *F* = *kd*, where *k* is the cantilever’s spring constant and *d* is the measured deflection of the cantilever. The spring constant k was calibrated using the thermal noise method (“thermal tune” application of the AFM software) and was found to be *k* = 0.034 N/m. The indentation depth *h* was calculated from the difference between the *z* movement of the piezoelectric motor and the deflection *d* of the cantilever. Hertz’s contact model relates the total elastic force exerted by a pyramidal indenter (*F*) to the indentation depth (*h*) and Young’s Modulus (*E*). The relationship is given by [115]

$$F(h)=\frac{4E\tan(\alpha )}{(1-v^{2})\pi^{3/2}}h^{2}$$ (1)

Where α is the effective half angle of the pyramid, and ν is the sample’s Poisson ratio (ν ≈ 0.5 for incompressible materials such as the cell cytoplasm). In accordance with Eq 1, a second-order polynomial fit was generated for the approaching curve to retrieve E. A set of 20 points from each cell image was selected using the “point-and-shoot” application, and a minimum of 10 force curves were obtained from each point. All force curves were fitted to a second-order polynomial. The correlation coefficient (r^2^) was calculated for each fit, and those lower than 0.99 were discarded. A normalized histogram for Young’s Modulus was constructed and fitted to a Gaussian distribution. Data points outside the 95% confidence interval $\left(\bar{E}\pm 2\sigma \right)$ were discarded, and the histogram and Gaussian fit were recalculated. A Student’s t-test was used for comparison between groups, and in all cases, a p<0.05 was considered significant. Three independent experiments were performed in triplicate (*n* = 9), and at least ten cells per experiment were analysed.

### Study of Lamin A/C methylation in neuroblastoma patients

Raw data from DNA methylation profiling of primary neuroblastomas for 105 patients based on a 450K human methylation (dataset GSE73515 at gene expression omnibus https://www.ncbi.nlm.nih.gov/geo/)used in Heinrich et al study [116] was downloaded and analyzed using ADMIRE [117] with a customized genomic regions file, targeting only chr1:156083251-156109330(Human genome version hg19). Probes with a detection p-value larger than 0.01, as well as probes that failed in more than 30% of cases were excluded. Remaining probes were normalized using a quantile normalization as suggested in [118].

The Q-value cutoff for multiple testing was set to 0.05. In the region under investigation, 25 probes passed the quality assessment. Within the accessed promoter region of -37 to +333 to Lamin A/C TSS location, three probes were valid (cg23922289, cg20018234, cg04317543). Normalized beta values were visualized via Bioconductor R package Complex Heatmap with hierarchical clustering on patients (columns) (https://bioconductor.org/packages/release/bioc/html/ComplexHeatmap.html).

### Statistical analysis

Student's *t* test was used for statistical comparison between two groups. If there were more than two groups, we used the one-way ANOVA test. The analysis was performed with Graph Pad Prism 7 Software (GraphPad Software, Inc., La Jolla, CA, USA).

## Supporting information

S1 Table Characteristics of patients with neuroblastoma according to the methylation status of cg08881019 and cg03946955 (region 2). (DOCX)
