# Supplementary material for: Promoter hypermethylation as a mechanism for Lamin A/C silencing in a subset of neuroblastoma cells
Source: PLoS One. 2017 Apr 19;12(4):e0175953. doi: 10.1371/journal.pone.0175953 (PMC5397038; doi:10.1371/journal.pone.0175953)
Supplement: S1 Table — (DOCX) [file pone.0175953.s001.docx]

| **Risk** | **Methylation status** | | **Total** | |
| --- | --- | --- | --- | --- |
|  | Unmethylated | Methylated |  | |
| Low | 26 | 14 | **40** | |
| Intermediate | 4 | 5 | **9** | |
| High | 24 | 32 | **56** | |
| **Total** | **54(51.4%)** | **51(48.6%)** | **105** | |
|  |  |  |  | |
| **Inss_stage** |  |  |  | |
| 1 | 7 | 3 | 10 | |
| 2.1 | 3 | 2 | 5 | |
| 2.2 | 2 | 2 | 4 | |
| 3 | 3 | 7 | 10 | |
| 4 | 27 | 29 | 56 | |
| 4S | 12 | 8 | 20 | |
| **Total** | **54(51.4%)** | **51(48.6%)** | | **105** |
